# Research Landscape of Chronic Pain and Neuropsychiatric Symptoms (1983–2025): A Comprehensive Bibliometric Analysis

**DOI:** 10.1155/prm/8816098

**Published:** 2026-11-01

**Authors:** Xingpu Quan, Xinrui Fu, Tao Lu, Jiandong Lai, Jingling Qiang, Xin Chen, Siyuan Chen, Ruiting Zhu, Ming Zhang, Xuan Niu

**Affiliations:** ^1^ Department of Medical Imaging The First Affiliated Hospital of Xi’an Jiaotong University Xi’an China xjtu.edu.cn; ^2^ Xi’an Jiaotong University Health Science Center Xi’an China xjtu.edu.cn; ^3^ Medical Imaging Center Ankang Central Hospital Ankang China akzxyy.com; ^4^ School of Life Science and Technology Xi’an Jiaotong University Xi’an China xjtu.edu.cn; ^5^ Department of Neurosurgery Yan’an University Affiliated Hospital Yan’an China yauhosp.com/; ^6^ School of Future Technology Xi’an Jiaotong University Xi’an China xjtu.edu.cn

**Keywords:** bibliometric analysis, chronic pain, depression, neuropsychiatric symptoms, pain management

## Abstract

**Objectives:**

Chronic pain affects a substantial proportion of the global adult population and is closely associated with neuropsychiatric symptoms, such as depression and anxiety, which may further worsen pain outcomes and increase mortality risk. However, development trends and research hotspots in this field remain insufficiently clarified. This study aimed to provide a macroscopic overview of research on the relationship between chronic pain and neuropsychiatric symptoms and to identify future directions for research and clinical practice, with particular attention to interdisciplinary collaboration and personalized treatment strategies.

**Methods:**

A total of 3627 publications between 1983 and 2025 were included in the final analysis. Bibliometric analyses and visualizations were performed using the R‐based Bibliometrix package, VOSviewer, CiteSpace, and Microsoft Excel 2019.

**Results:**

The number of publications in this field showed a steady upward trend, with rapid growth after 2017. Among contributing countries, the United States emerged as the most active contributor and demonstrated the strongest international collaboration. Leading institutions, including Harvard University; high‐impact journals, such as *Neurology* and *Headache*; and influential researchers, such as Lipton RB, made prominent contributions to the field. Keyword co‐occurrence, citation burst, and thematic evolution analyses indicated that “chronic pain,” “pain management,” “migraine,” “depression,” and “anxiety” remained central research themes, whereas “long COVID,” “mental health,” and “quality of life” may represent emerging future research directions.

**Conclusion:**

Research in this field is increasingly shifting toward integrated pain management that incorporates neuropsychiatric and psychosocial dimensions. Future academic and clinical efforts should prioritize the development of individualized treatment frameworks to optimize patient outcomes and refine existing pain management protocols.

## 1. Introduction

Chronic pain, defined by the International Association for the Study of Pain (IASP) as persistent or recurrent pain lasting longer than three months, remains a major burden on global public health systems [1]. According to the American Pain Society Pain Taxonomy, this condition encompasses diverse categories, such as musculoskeletal, visceral, pelvic/urogenital, and both peripheral and central neuropathic pain [2]. Epidemiological data from the United States indicate that chronic pain affects approximately 11%–40% of adults [3]. In addition to increasing healthcare utilization, chronic pain is closely associated with reduced quality of life and a substantial economic burden [4]. Although therapeutic strategies have continued to evolve, many clinical guidelines remain focused on broad pain symptoms, and the implementation of personalized or precision medicine remains challenging in current practice [5].

Neuropsychiatric symptoms are among the most common and disabling comorbid manifestations experienced by patients with chronic pain [6, 7]. These symptoms encompass a broad spectrum of emotional, cognitive, and somatic manifestations, including depression, anxiety, impaired concentration, cognitive dysfunction, fatigue, and insomnia [8, 9]. Previous studies have suggested that 30%–45% of patients with chronic pain experience comorbid conditions, such as depression, anxiety, or obsessive‐compulsive symptoms, and that the prevalence of these symptoms may increase with pain chronicity [10]. These psychiatric comorbidities are significant predictors of poor clinical prognosis, including heightened pain sensitivity, increased functional disability, and elevated mortality risk. From a mechanistic perspective, the bidirectional relationship between chronic pain and neuropsychiatric symptoms may involve autonomic nervous system dysregulation, structural reductions in gray matter volume, and increased levels of pro‐inflammatory cytokines [11]. However, conventional treatments, such as antidepressants [12], cognitive‐behavioral therapy [13], and physical therapy [14], may provide incomplete relief for patients with complex comorbid symptoms, underscoring the need for a deeper understanding of the interplay between chronic pain and neuropsychiatric health.

Bibliometric analysis is a widely used quantitative method for mapping research trends, knowledge structures, influential contributors, and emerging topics within a specific field [15]. It has also been applied to chronic pain research, with several studies exploring associations between pain and comorbid neuropsychiatric symptoms, such as anxiety and depression [16, 17]. However, previous studies have primarily focused on one or two specific neuropsychiatric symptoms, thereby providing limited insight into the broader relationship between chronic pain and neuropsychiatric symptoms.

In this study, we conducted a bibliometric analysis to examine the global research landscape of chronic pain and neuropsychiatric symptoms from 1983 to 2025. We aimed to map the primary knowledge structure of this field, identify major contributors and collaboration patterns, and explore how research themes have evolved over more than four decades. These findings may provide a comprehensive overview of the current evidence and offer a theoretical foundation for future research and clinical strategies targeting patients with chronic pain and comorbid neuropsychiatric symptoms.

## 2. Methods

### 2.1. Data Collection and Search Strategy

A comprehensive literature search was conducted in the Web of Science Core Collection (WoSCC), Scopus, and PubMed. The time span ranged from January 1, 1983, to December 31, 2025. To minimize bias associated with frequent database updates, all searches and data retrieval were completed on the same day, May 5, 2026. The detailed search strategies for WoSCC, Scopus, and PubMed are provided in the Supporting Information.

### 2.2. Publication Inclusion and Exclusion Criteria

Publications related to chronic pain and neuropsychiatric symptoms were included if they were original research articles, reviews, or case reports. The exclusion criteria were as follows: (i) book chapters, guidelines, corrections, editorial materials, and proceeding papers; (ii) duplicate publications; and (iii) non‐English publications. After database‐specific exclusions, 2369 records from WoSCC, 2112 records from Scopus, and 1136 records from PubMed were retained.

### 2.3. Data Export and Integration

After database‐specific document screening according to the predefined eligibility criteria, records were exported separately from each database. Records from WoSCC were exported in plain‐text format using the “Full Record and Cited References” option; records from Scopus were exported in CSV format; and records from PubMed were exported in Citation Manager (.nbib) format. Because of the Scopus platform’s export limits, records were exported in two separate files, which were imported separately and combined before cross‐database integration. All exported records were subsequently imported into R for bibliographic preprocessing and integration using the Bibliometrix package (Version 5.3.0).

The convert2df() function in Bibliometrix was used to convert the WoSCC, Scopus, and PubMed files into separate bibliographic data frames. Before cross‐database integration, core bibliographic fields were harmonized across databases, including authors, article titles, source or journal names, publication years, digital object identifiers (DOIs), abstracts, keywords, institutional affiliations, document types, and cited references. DOI strings were normalized by converting letters to lowercase, removing URL or DOI prefixes, and eliminating extra spaces and unnecessary terminal punctuation. Article titles were standardized for capitalization, punctuation, and repeated spaces. Author and source fields were checked for differences in delimiters, capitalization, and spacing, and publication years were converted into a consistent four‐digit format. Author keywords, indexed keywords, and MeSH terms were mapped to comparable keyword variables while retaining the original database‐specific fields. Because WoSCC, Scopus, and PubMed differ in indexing systems and field structures, semantically equivalent fields were mapped to a common set of unified variables, while the original database‐specific fields were preserved to maintain data integrity.

### 2.4. Deduplication and Manual Verification

Duplicate records were identified and removed using a combination of automated deduplication and manual verification. Automated deduplication was performed using the mergeDbSources() function with the remove.duplicated = TRUE option after field harmonization. Records with valid DOI information were checked primarily by normalized DOI. For records without DOI information or with inconsistent DOI values, normalized article title and publication year were used as secondary matching criteria. Records that remained unresolved were further evaluated using normalized title, first author, and publication year.

During manual verification, the complete article title, author list, source or journal name, publication year, volume, issue, page range, and other available bibliographic information were considered jointly. This procedure was designed to minimize both the retention of duplicate records and the erroneous merging of distinct publications with similar titles. WoSCC and Scopus records were first merged, with 1017 duplicates removed; PubMed records were then added, and a further 973 duplicates were removed. Finally, 3627 publications were included in the bibliometric analysis. The screening and selection procedures were conducted independently by two researchers. In cases of disagreement, a third researcher was consulted to make the final decision. The detailed flowchart of the publication selection process is shown in Figure 1.

**FIGURE 1:**
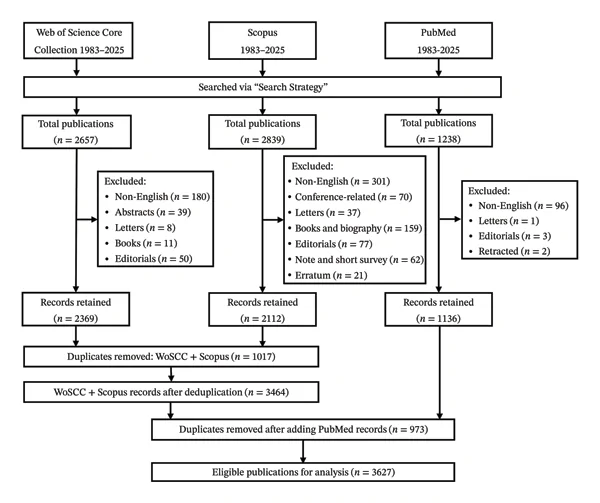
Flowchart of the publication selection process.

### 2.5. Bibliometric Analysis

The deduplicated combined dataset from WoSCC, Scopus, and PubMed was used for descriptive analyses, including annual publication output and overall publication counts. Records with available bibliographic information were included in country/region, institution, source, and keyword analyses. Citation analyses and cited‐reference analyses were conducted using WoSCC and Scopus records because these databases provide more complete and standardized citation counts and cited reference information.

The R‐based Bibliometrix package (Version 5.3.0) was used to analyze annual publication output, visualize collaborative networks among countries, and perform thematic evolution analysis based on keyword data. Temporal changes in publication output were quantitatively assessed using the compound annual growth rate (CAGR) and exponential growth modeling of log‐transformed annual publication counts to characterize the overall long‐term growth pattern. Segmented linear regression was then performed to identify major transition points in annual publication output and to define distinct publication‐growth phases. Candidate breakpoint models were compared using the Bayesian information criterion (BIC), and the estimated slope for each phase was used to quantify the annual increase in publication output. For thematic evolution analysis, the study period was divided into two time slices: 1983–2016 and 2017–2025. Although segmented trend analysis identified multiple publication‐growth phases, 2017 was selected as the thematic cutoff because it represented the transition point into the steepest growth phase. Bradford’s law was applied to identify core journals by categorizing sources into zones according to publication productivity [18].

VOSviewer (Version 1.6.20) was used to visualize bibliometric networks, including institutional collaboration, keyword co‐occurrence, cocited authors, and cocited sources. Node selection was performed during preprocessing: The institutional collaboration network included the 60 most productive institutions; the cocited author and cocited source networks included the top 100 items by citation counts; and the keyword co‐occurrence network included the top 100 keywords by occurrence frequency. Association strength was used as the VOSviewer normalization method.

CiteSpace (Version 7.0.R0) was used to identify keywords with the strongest citation bursts, including their time intervals and durations, to highlight emerging research topics. For CiteSpace analysis, the time span was set from 1983 to 2025, with one year per slice. Burst detection was performed with the gamma (*γ*) value set to 0.5, and the remaining parameters were kept at the default settings. Microsoft Excel 2019 was used to generate figures illustrating annual publication output and institutional publication counts.

## 3. Results

### 3.1. Quantity and Trend Analysis of Annual Publications

A total of 3627 papers in the field of chronic pain and neuropsychiatric symptoms were identified. Annual publication output increased from 3 articles in 1983 to 334 articles in 2025, corresponding to an overall CAGR of 11.9%. The exponential growth model fitted to log‐transformed annual publication counts showed a strong fit (*R* ^2^ = 0.937), supporting sustained long‐term growth in research activity. Segmented linear regression further identified three major transition points in annual publication output: 2000, 2017, and 2020 (Figure 2 and Supporting Table 1). Accordingly, the publication trajectory was divided into four quantitatively defined phases: Phase I (1983–2000), Phase II (2001–2017), Phase III (2018–2020), and Phase IV (2021–2025). The estimated annual increase in publication output rose from 1.6 articles/year in Phase I to 6.3 articles/year in Phase II and 31.5 articles/year in Phase III, followed by continued high‐level publication output in Phase IV with an estimated slope of 17.6 articles/year. These findings indicate a marked acceleration of research activity after 2017, followed by sustained high publication output from 2021 onward.

**FIGURE 2:**
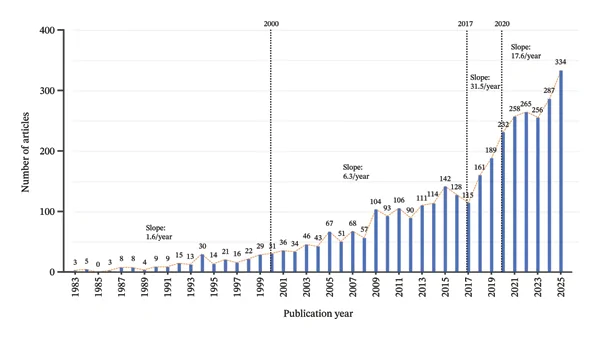
Annual scientific production in the field of chronic pain and neuropsychiatric symptoms from 1983 to 2025. Vertical dotted lines indicate the transition points identified by segmented linear regression. The estimated slopes for each phase are shown in the figure.

### 3.2. Analysis of Countries and Institutions

Figure 3A illustrates the global country collaboration network. International collaborations were widely distributed across North America, Europe, Asia, and Australia. The strongest collaborative links were observed between the United States and several countries, including the United Kingdom, Canada, and Germany (Table 1). Figure 3B shows the distribution of corresponding author countries. The United States contributed the largest number of publications (*n* = 1103), followed by the United Kingdom (*n* = 194), Italy (*n* = 164), China (*n* = 142), and Germany (*n* = 136). The United States also had the highest numbers of both single‐country publications (SCP = 978) and multiple‐country publications (MCP = 125). Although China ranked fourth in total publications, its output predominantly consisted of SCP (SCP = 135; MCP = 7).

**FIGURE 3:**
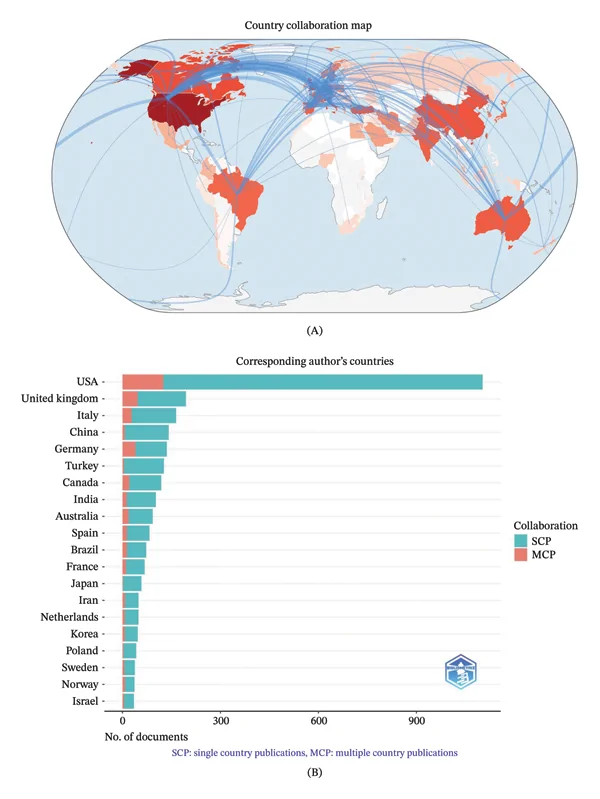
The most influential countries. (A) Country collaboration map. Color intensity reflects the number of publications from each country, and curved lines represent collaborative links between countries. (B) Distribution of corresponding author countries. Countries are ranked by total publication count, with bars divided into single‐country publications (SCP) and multiple‐country publications (MCP).

**TABLE 1:** Top 10 productive countries and major international collaborations.

Rank	Country	Articles	SCP	MCP	Rank	Collaboration	Frequency
1	USA	1103	978	125	1	USA–United Kingdom	47
2	United Kingdom	194	147	47	2	USA–Canada	45
3	Italy	164	136	28	3	USA–Germany	43
4	China	142	135	7	4	Germany–United Kingdom	32
5	Germany	136	96	40	5	USA–Italy	29
6	Turkey	127	122	5	6	Germany–Italy	27
7	Canada	119	98	21	7	USA–Australia	27
8	India	102	89	13	8	United Kingdom–Spain	26
9	Australia	93	74	19	9	USA–Brazil	25
10	Spain	83	69	14	10	United Kingdom–Australia	24

Abbreviations: MCP, multiple‐country publications; SCP, single‐country publications; USA, United States of America.

Figure 4 shows the 20 most productive institutions and the institutional collaboration network. Harvard University ranked first with 278 publications, followed by Harvard Medical School (*n* = 220), the University of London (*n* = 150), the University of Toronto (*n* = 149), and the University of Pennsylvania (*n* = 134) (Figure 4A). The institutional collaboration network revealed a dense collaborative core centered on major North American institutions, particularly Harvard University, Massachusetts General Hospital, and the University of California System. European institutions, including the University of London, King’s College London, Université Paris Cité, and the University of Oslo, formed another major collaborative cluster and maintained links with the North American network. Compared with North American and European institutions, Asian institutions were less prominent among the major nodes in the institutional collaboration network shown in Figure 4B.

**FIGURE 4:**
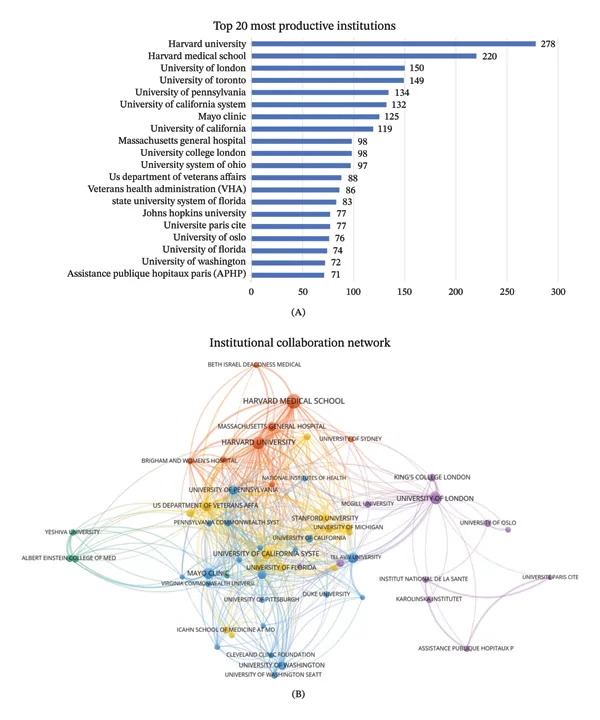
Institutional contribution and collaboration network. (A) Top 20 most productive institutions ranked by publication count. (B) Institutional collaboration network. Each node represents an institution, with node size reflecting publication output. Links indicate collaborative relationships, with thicker links representing stronger collaboration. Colors denote collaboration clusters.

### 3.3. Analysis of Authors and Cocited Authors

Table 2 presents the 10 authors with the highest H‐index values, together with their publication and citation counts. The H‐index is a widely used scientometric indicator that reflects both an author’s publication output and citation impact [19]. Among the listed authors, Lipton RB ranked first, with the highest H‐index (13), the largest number of publications (*n* = 20), and the highest citation count (*n* = 1510). Dodick DW ranked second with an H‐index of 12, followed by Wang SJ with an H‐index of 10. The cocited author network (Figure 5A) further reveals the intellectual structure through several highly interconnected clusters. Silberstein SD, Olesen J, Diener HC, and Bigal ME were closely interconnected, forming a prominent cocitation cluster. In addition, Wolfe F, Kessler RC, Beck AT, Lipton RB, and Goadsby PJ appeared as prominent nodes in other major clusters. Notably, Lipton RB and Goadsby PJ were included in both the H‐index ranking and the cocitation network, suggesting that these highly productive authors also had strong cocitation prominence in this field.

**TABLE 2:** The top 10 authors ranked by H‐index.

Rank	Authors	H‐index	Publications	Citations
1	Lipton RB	13	20	1510
2	Dodick DW	12	16	578
3	Wang SJ	10	19	942
4	Goadsby PJ	8	11	769
5	Kim J	8	15	360
6	Lee J	8	18	348
7	Wang J	8	20	214
8	Fregni F	7	17	1053
9	Kim H	7	10	222
10	Kim S	7	12	256

**FIGURE 5:**
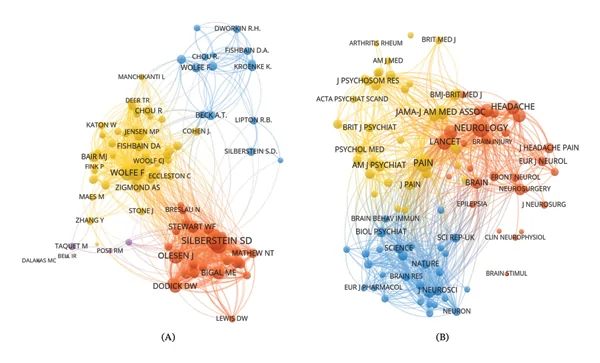
Cocitation networks of authors and journals. (A) Cocited author network. Each node represents a cited author, node size indicates citation frequency, links represent cocitation relationships, and colors denote cocitation clusters. (B) Cocited journal network. Each node represents a cited journal, node size indicates citation frequency, links represent cocitation relationships, and colors denote journal clusters.

### 3.4. Analysis of Journals and Cocited Journals

Table 3 presents the 10 journals with the highest H‐index, which provides a more comprehensive assessment of a journal’s long‐term scientific or clinical influence than traditional impact factors [20]. *Neurology* ranked first, with an H‐index of 41, 92 publications, and 4612 citations. *Headache* ranked second, with an H‐index of 24, 77 publications, and 2808 citations. *Journal of Clinical Psychiatry*, *Pain Medicine*, and *Pain* were also among the leading journals. Bradford’s law was applied across all identified journals to examine the distribution of source journals by publication productivity and identify core journals in the field [21]. The journals were divided into three Bradford zones, with 66 journals in the core zone, 333 journals in Zone 2, and 1049 journals in Zone 3. The Bradford distribution and the top 10 journals ranked by publication output are shown in Supporting Figure S1.

**TABLE 3:** The top 10 journals ranked by H‐index.

Rank	Journals	H‐index	Publications	Citations
1	Neurology	41	92	4612
2	Headache	24	77	2808
3	Journal of Clinical Psychiatry	18	27	1033
4	Pain Medicine	18	33	697
5	Pain	15	29	1653
6	European Journal of Paediatric Neurology	14	31	569
7	European Journal of Neurology	12	26	686
8	Journal of Headache and Pain	12	27	308
9	PLOS ONE	11	21	413
10	Biological Psychiatry	10	19	1966

The cocited journal network (Figure 5B) further illustrates citation relationships among major journals. High‐impact journals, such as *Pain*, *Neurology*, and *The Lancet*, appeared as prominent nodes. *Neurology*, *Headache*, *The Lancet Neurology*, *Brain, and European Journal of Neurology* were closely linked, suggesting a strong citation base related to neurological mechanisms, headache disorders, and clinical advances. Another closely interconnected cluster included *Pain, JAMA (Journal of the American Medical Association), Journal of Psychosomatic Research,* and *American Journal of Psychiatry*, reflecting the integration of pain research with psychological factors, mental health, and psychosomatic medicine.

### 3.5. Analysis of Article Citations

Table 4 presents the 10 most influential documents ranked by local citations (LC). LC refers to the number of citations a document receives from other publications within the retrieved dataset, reflecting its influence within the specific research corpus. In contrast, global citations (GC) refer to the number of citations a document receives in the source database, indicating broader citation impact [22]. As shown in Table 4, the article by Scher AI, published in *Headache* in 1998, ranked first, with the highest LC count (*n* = 19) and a GC count of 440. The article by Castillo J, published in *Headache* in 1999, ranked second, with an LC count of 14 and a GC count of 444. Notably, the article by McWilliams LA, published in *Pain*, had the highest GC count among the 10 documents (*n* = 637), suggesting that this article had substantial broader citation impact beyond its local influence within the retrieved dataset.

**TABLE 4:** The top 10 documents ranked by local citations.

No.	Document	DOI	LC	GC	LC/GC (%)	Normalized	Normalized
						LC	GC
1	Scher AI, 1998, Headache	10.1046/j.1526‐4610.1998.3807497.x	19	440	4.32	19.00	5.55
2	Castillo J, 1999, Headache	10.1046/j.1526‐4610.1999.3903190.x	14	444	3.15	13.50	8.91
3	McWilliams LA, 2003, Pain	10.1016/S0304‐3959(03)00301‐4	10	637	1.57	13.87	8.79
4	Juang KD, 2000, Headache	10.1046/j.1526‐4610.2000.00148.x	10	222	4.50	12.50	3.92
5	Bigal ME, 2008, Neurology	10.1212/01.wnl.0000335946.53860.1d	9	203	4.43	17.68	6.48
6	Sampson EL, 2015, Pain	10.1097/j.pain.0000000000000095	8	131	6.87	18.69	5.81
7	Robbins MS, 2010, Neurology	10.1212/WNL.0b013e3181dad5de	8	94	8.51	16.36	3.03
8	Schrag A, 2004, Brain	10.1093/brain/awh262	7	224	3.12	7.97	5.89
9	Fink P, 2010, J Psychosom Res	10.1016/j.jpsychores.2010.02.004	6	228	2.63	12.27	7.36
10	Wang SJ, 2001, Pain	10.1016/S0304‐3959(00)00380‐8	6	145	4.14	9.71	4.28

Abbreviations: GC, global citations; LC, local citations.

### 3.6. Analysis of Keyword Co‐Occurrence and Citation Bursts

The keyword co‐occurrence network visualizes the relationships among keywords through links and clusters, with node size reflecting keyword occurrence frequency (Figure 6A). Using unsupervised clustering with VOSviewer, the keyword co‐occurrence network was categorized into five thematic clusters: Cluster 1, clinical pain management and rehabilitation; Cluster 2, neurological disorders, neuroinflammation, and cognitive impairment; Cluster 3, psychiatric symptoms and fatigue‐related conditions; Cluster 4, headache disorders; and Cluster 5, COVID‐19‐related neuropsychiatric research. The most prominent keywords in the network included “chronic pain,” “pain,” “migraine,” “neurology,” “pain management,” “dementia,” and “depression.”

FIGURE 6Keyword and thematic analyses of neuropsychiatric symptoms in chronic pain. (A) Keyword co‐occurrence network showing major research topics. Node size indicates keyword frequency, links represent co‐occurrence relationships, and colors indicate keyword clusters. (B) Top 20 keywords with the strongest citation bursts. Red bars indicate burst periods, and strength reflects burst intensity. (C) Thematic map of research themes from 1983 to 2016. (D) Thematic map of research themes from 2017 to 2025.

**Figure:**
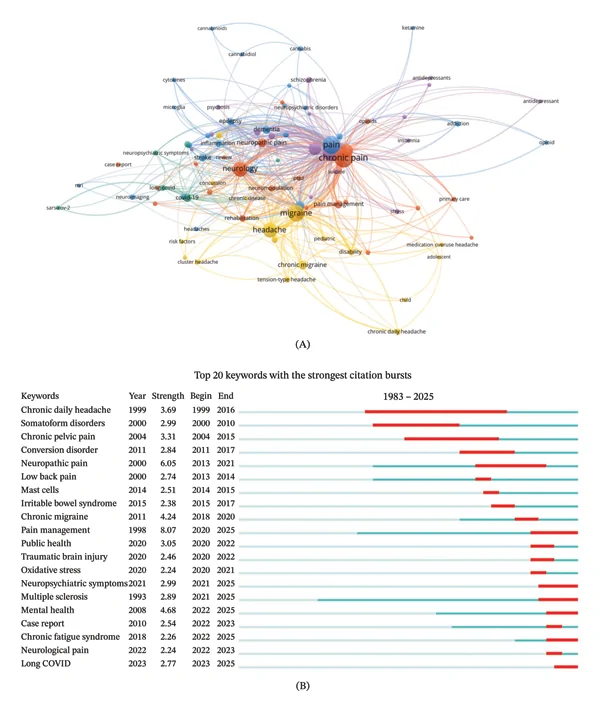

**Figure:**
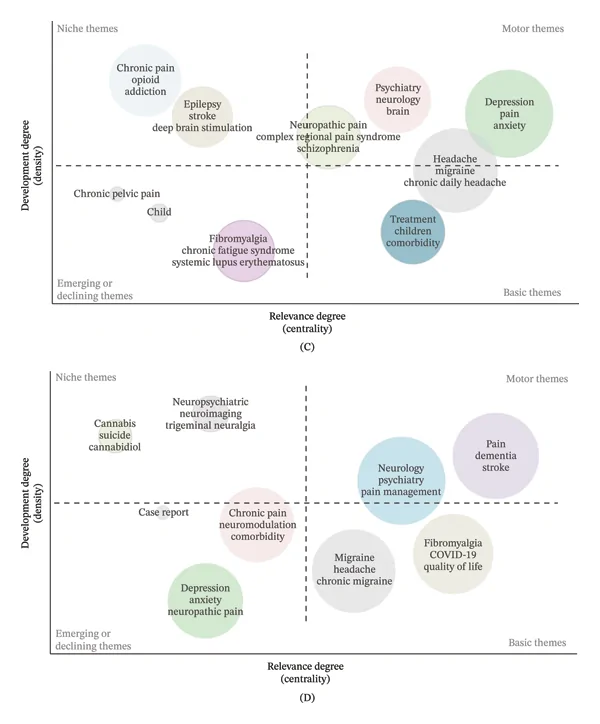

Figure 6B shows the 20 keywords with the strongest citation bursts, referring to terms that received sharply increased attention during specific time intervals [23]. Earlier keywords with citation burstsincluded “chronic daily headache,” “somatoform disorders,” “chronic pelvic pain,” “conversion disorder,” “neuropathic pain,” and “low back pain,” suggesting sustained attention to specific chronic pain conditions. In addition, keywords, such as “pain management,” “multiple sclerosis,” “neuropsychiatric symptoms,” “mental health,” “chronic fatigue syndrome (CFS),” and “long COVID,” exhibited ongoing bursts through 2025, which may indicate emerging future research trends.

### 3.7. Analysis of Thematic Evolution

Based on the annual publication trend shown in Figure 2, 2017 was used as the cutoff year for the thematic evolution analysis. Figure 6C,D illustrate thematic maps of research themes for the periods 1983–2016 and 2017–2025, respectively. A two‐dimensional map was constructed based on thematic centrality (relevance) and density (development), dividing themes into four quadrants: motor themes (high density, high centrality), basic themes (low density, high centrality), niche themes (high density, low centrality), and emerging or declining themes (low density, low centrality) [24]. From 1983 to 2016, “depression,” “anxiety,” “psychiatry,” and “neurology” were located in the motor themes quadrant. From 2017 to 2025, “dementia,” “stroke,” and “pain management” emerged as motor themes, suggesting increasing attention to the intersection of chronic pain, cognitive impairment, and pain management. In the later period, newly emerging basic themes included “migraine,” “fibromyalgia,” “COVID‐19,” and “quality of life,” suggesting that these topics may continue to develop in future research. In contrast, themes such as “cannabis,” “neuromodulation,” and “case reports” were located in the niche or emerging or declining quadrants, reflecting reduced sustained attention to these topics.

## 4. Discussion

This bibliometric analysis provides a comprehensive overview of research progress on chronic pain and neuropsychiatric symptoms over more than four decades. By integrating records from WoSCC, Scopus, and PubMed, the present study characterized publication‐growth patterns, collaboration networks, influential sources, keyword bursts, and thematic evolution, thereby identifying emerging frontiers and remaining knowledge gaps to guide future research directions.

Previous bibliometric studies have provided valuable insights into pain‐related topics. For example, studies on neuropathic pain associated with depression or anxiety have reported rapid growth in this focused comorbidity area [25, 26]. Recent bibliometric analyses have also mapped emerging topics, such as migraine treatment, fibromyalgia, cognitive‐behavioral therapy, and technological applications in chronic pain research [27–30]. However, these studies have generally focused on specific pain disorders, selected psychiatric comorbidities, or particular therapeutic and methodological domains. In contrast, the present study provides a broader overview of the intersection between chronic pain and neuropsychiatric symptoms by integrating evidence from multiple databases over more than four decades. Beyond describing publication volume, the combination of growth modeling, keyword bursts, and thematic evolution helps trace how this field has gradually moved from a primarily pain‐centered perspective toward a multidimensional framework involving psychiatric comorbidity, cognitive symptoms, fatigue, sleep disturbance, quality of life, and neurological comorbidity.

Analysis of countries and institutions indicates that the United States, particularly Harvard University, is a major contributor and collaborator in this field. Studies from Harvard University have focused on behavioral interventions for chronic pain and insomnia, noninvasive brain stimulation, and clinical guidelines for diabetic painful polyneuropathy, reflecting sustained attention to pain management, neural mechanisms, and pain‐related public health issues [31, 32]. Although China had a number of SCPs comparable to those of Italy and Germany, its number of MCPs remains limited, suggesting that its research output was driven more by domestic efforts than by international collaboration. Overall, these findings indicate an uneven global collaboration pattern, with research productivity and international cooperation still concentrated in North America and Europe. Future efforts should prioritize fostering closer collaboration between established research institutions in North America and Europe and emerging research groups in Asia and other developing regions.

This study further examined author productivity, journal influence, and the citation structure of this field. Lipton RB, the author with the highest H‐index, has contributed substantially to migraine epidemiology and risk factor research, highlighting the high prevalence, underdiagnosis, socioeconomic burden, and psychiatric comorbidity of migraine [33–35]. The highly cited studies within the dataset suggest that the knowledge base of this field has extended beyond epidemiological investigation toward refined clinical classification, comorbidity recognition, and comprehensive assessment of patient outcomes [36, 37]. By linking chronic pain with psychiatric comorbidity and impaired quality of life, these studies have helped build an evidence base for improving clinical diagnostic frameworks and developing integrated intervention strategies [38, 39]. At the journal level, *Neurology* and *Headache* ranked among the leading sources, while *Pain*, *The Lancet*, and other major journals were frequently identified in the cocited source network, further suggesting that this research area is positioned at the intersection of pain medicine, neurology, psychiatry, and broader clinical medicine. These findings may help researchers identify key knowledge sources and promote more interdisciplinary publication and collaboration strategies.

Quantitative growth analysis showed a sustained increase in publication output, with segmented linear regression identifying 2000, 2017, and 2020 as major transition points. During the early stage, research output remained low, possibly reflecting limited epidemiological evidence and the absence of widely accepted clinical protocols [40]. The gradual increase in publication output after 2000 may be related to broader recognition of pain management as a public health priority [41]. Notably, the transition point in 2017 marked the beginning of a more rapidly expanding phase, which may be associated with the systematization of diagnostic and clinical management frameworks for chronic pain, including the IASP classification of chronic pain for ICD‐11, as well as increasing research on psychiatric and neuropsychiatric comorbidities, such as depression, anxiety, sleep disturbance, fatigue, and cognitive symptoms [1, 42]. Consistently, the later thematic map further indicated that research after 2017 became more structurally diversified, with greater attention to pain management, neurological diseases, such as dementia and stroke, and quality of life. After 2020, publication output remained at a high level despite short‐term fluctuations, possibly reflecting the impact of public health issues, such as COVID‐19 and growing attention to postinfectious symptom burden [43]. Recent citation bursts of “public health,” “mental health,” “chronic fatigue syndrome,” and “long COVID” further support this interpretation, indicating that public health events, postinfectious symptom burden, and related mental health effects are becoming increasingly important clinical contexts for chronic pain research and pain management.

These changes also place greater emphasis on how chronic pain is managed in increasingly complex clinical contexts. Consistently, “pain management” demonstrated an ongoing citation burst and appeared as a motor theme in the later period. Comprehensive pain management is a multidisciplinary approach aimed at alleviating pain, improving physical and psychological functioning, and enhancing quality of life for individuals experiencing acute or chronic pain [7, 44]. The IASP working group recommends that interdisciplinary pain treatment centers include a broad range of medical specialists with substantial expertise to comprehensively address the biopsychosocial model of pain. However, most pain professionals practice as independent clinicians, and implementation of interdisciplinary care often encounters procedural and financial barriers [45]. In addition, current approaches and guidelines typically focus on specific pain conditions or symptoms, and personalized or precision medicine for chronic pain remains challenging [5, 46]. Nonpharmacological interventions, including cognitive‐behavioral therapy and noninvasive brain neuromodulation, may provide short‐term benefits for chronic pain management. However, their long‐term effectiveness across different pain conditions, pain mechanisms, and psychiatric comorbidity profiles remains uncertain, partly because of heterogeneous treatment protocols, variable patient selection criteria, inconsistent outcome measures, and limited long‐term evidence [47]. Future research should clarify which patients benefit from specific interventions, develop structured psychological intervention protocols, reduce overreliance on pharmacological therapies where appropriate, strengthen multidisciplinary collaboration, promote pain rehabilitation, and advance the standardization of chronic pain management.

Postinfectious and multisystem conditions are becoming increasingly relevant to chronic pain research, particularly in relation to long COVID and CFS. Long COVID is a heterogeneous clinical syndrome characterized by persistent symptoms, such as widespread pain, fatigue, cognitive difficulties, sleep disturbances, and psychological distress [48]. Current studies have proposed multiple potentially interacting mechanisms and contributing factors, including immune dysregulation, neuroinflammation, vascular dysfunction, gut‐brain axis alterations, central sensitization, and psychosocial stress [49]. Persistent COVID‐related headaches during the COVID‐19 pandemic may have reflected the combined influence of biological factors, pandemic‐related stress, and psychological burden [50]. Although the acute phase of the pandemic has passed, its long‐term effects on pain, fatigue, cognition, and mental health remain clinically important [51]. Similarly, CFS is commonly characterized by chronic disabling fatigue, impaired concentration, muscle and joint pain, and other somatic symptoms [52]. The mechanisms underlying increased pain experiences in CFS remain incompletely understood, but current evidence suggests possible involvement of central sensitization, generalized low‐grade inflammation, and emotional and cognitive processes [53]. The relative contributions of infection‐related biological changes, persistent fatigue, associated sleep disturbance, activity limitation, and psychological burden to pain remain uncertain. Future studies should focus on longitudinal tracking of disease progression, standardized diagnostic criteria, biomarker assessment, and symptom evolution to better clarify the mechanisms and long‐term effects of these complex conditions on pain and neuropsychiatric health.

The emergence of “dementia” and “stroke” as motor themes in the later period also warrants attention. This finding suggests that pain research is increasingly extending to populations with cognitive impairment and neurological diseases [54]. Existing evidence suggests a potential association between pain and neuropsychiatric symptoms, but the directionality and underlying mechanisms remain unclear. Behavioral symptoms in dementia may be associated with pain‐related distress and may also be influenced by neurodegenerative changes, sleep disturbance, medication use, and environmental or care‐related conditions [55]. Clinically, pain is frequently underdetected in patients with dementia or communication difficulties after stroke, and untreated pain can exacerbate agitation, behavioral symptoms, sleep disturbances, depression, anxiety, and functional decline [55, 56]. Accurate assessment of pain in these populations remains challenging, as cognitive decline, aphasia, and impaired awareness may limit the reliability of self‐reported pain measures. Similarly, poststroke pain may interact with mood disorders, cognitive impairment, and rehabilitation outcomes [57, 58]. Potential mechanisms of central poststroke pain may involve the spinal‐thalamo‐cortical pathway and multiple neural signaling processes, but its pathogenesis remains incompletely understood [59]. Accordingly, future studies should develop reliable pain assessment tools and integrated care strategies for patients with cognitive or communication impairment, and longitudinal or interventional studies are needed to determine whether improved pain management can reduce behavioral symptoms and improve functional outcomes.

Several limitations of this study should be noted. First, the inclusion criteria may have excluded other potentially relevant publication types and non‐English literature, which could also provide valuable insights into this field. Second, citation‐based metrics may underestimate the impact of recently published articles, as they have had less time to accumulate citations and may therefore not fully reflect the influence of emerging studies or rapidly developing topics. Third, the clustering and visualization results generated by bibliometric tools, such as VOSviewer and CiteSpace, may be affected by software‐specific algorithms and analytical settings, and should therefore be interpreted as exploratory, data‐driven patterns rather than definitive representations of the field. Fourth, although the search strategy was carefully designed, neuropsychiatric symptoms remain an evolving research area, and some relevant publications may not have been fully captured. Despite these limitations, this study provides a comprehensive overview of the research landscape concerning neuropsychiatric symptoms in chronic pain and offers valuable insights into current trends and potential areas for future investigation.

## 5. Conclusion

This bibliometric analysis mapped the global research landscape of chronic pain and neuropsychiatric symptoms from 1983 to 2025 and identified sustained growth, major contributors, and evolving thematic priorities in this field. Clinically, these findings highlight the importance of comprehensive, multidimensional assessment and individualized, multidisciplinary management in patients with chronic pain. Future research should strengthen international and interdisciplinary collaboration, standardize the assessment of neuropsychiatric comorbidities, and further investigate the biological and psychosocial pathways linking chronic pain with neuropsychiatric symptoms. Overall, these efforts may help inform more targeted interventions and ultimately improve patient outcomes and quality of life.

## Author Contributions

Xingpu Quan and Xinrui Fu contributed equally to this work as co‐first authors. Xuan Niu supervised the study. Xingpu Quan, Xinrui Fu, and Tao Lu performed the data collection and statistical analyses and prepared the initial draft of the manuscript. Jiandong Lai, Jingling Qiang, Xin Chen, Siyuan Chen, Ruiting Zhu, and Ming Zhang contributed to data interpretation and participated in revising the manuscript.

## Funding

This work was supported by the following funding sources: the National Natural Science Foundation of China (Grant No. 82202121); the Shaanxi Provincial Young Star of Science and Technology Program (Grant No. 2025ZC‐KJXX‐125); the Health Research and Innovation Capacity Strengthening Platform Program of Shaanxi Province (Grant No. 2025PT‐06); the Key Industry Innovation Chain Fund in the Social Development Field of Shaanxi Province (Grant No. 2024SF‐ZDCYL‐01‐08); and the Xi’an Jiaotong University Free Exploration and Innovation Student Project (Grant No. xzy022025072).

## Disclosure

All authors reviewed and approved the final version of the manuscript.

## Ethics Statement

Ethics approval was not required for this study.

## Conflicts of Interest

The authors declare no conflicts of interest.

## Supporting Information

Additional supporting information can be found online in the Supporting Information section.

## Supporting information


**Supporting Information** Additional supporting information, including the detailed database search strategies, Supporting Table 1 on publication‐growth phases, and Supporting Figure S1 on Bradford’s Law analysis, is provided in the accompanying supporting file.

## Data Availability

The bibliographic data were retrieved from the WoSCC, Scopus, and PubMed. Access to WoSCC and Scopus may be subject to subscription or licensing restrictions, whereas PubMed records are publicly available. The processed data are available from the corresponding author upon reasonable request.
