# Supplementary material for: Research Landscape of Chronic Pain and Neuropsychiatric Symptoms (1983–2025): A Comprehensive Bibliometric Analysis
Source: Pain Res Manag. 2026 Nov 1;2026:8816098. doi: 10.1155/prm/8816098 (PMC13725059; doi:10.1155/prm/8816098)
Supplement: Supplementary file 1 [file PRM-2026-8816098-s001.docx]

**Supplementary Information**

**Methods**

**Search Strategy for Web of Science Core Collection (WoSCC)**

#1 TS=("chronic pain" OR "chronic primary pain" OR "chronic primary visceral pain" OR "chronic widespread pain" OR "chronic primary musculoskeletal pain" OR "chronic primary headache" OR "chronic primary orofacial pain" OR "chronic migraine" OR "chronic tension-type headache" OR "complex regional pain syndrome" OR "chronic cancer related pain" OR "chronic cancer pain" OR "chronic post cancer treatment pain" OR "chronic postsurgical pain" OR "chronic post traumatic pain" OR "chronic secondary musculoskeletal pain" OR "chronic secondary visceral pain" OR "chronic neuropathic pain" OR "chronic central neuropathic pain" OR "chronic peripheral neuropathic pain" OR "chronic secondary headache" OR "chronic secondary orofacial pain" OR "chronic dental pain" OR "chronic neuropathic orofacial pain" OR "persistent headache" OR "unspecified chronic pain" OR "fibromyalgia" OR "intractable pain" OR "chronic neck and shoulder pain" OR "chronic back pain" OR "chronic pelvic pain" OR "chronic pelvic pain syndrome" OR "chronic low back pain" OR "chronic spine pain" OR "chronic limb pain" OR "urologic chronic pelvic pain syndrome" OR "persistent postoperative pain" OR "widespread chronic pain" OR "chronic orofacial pain" OR "persistent pain" OR "continuous pain" OR "recurrent pain" OR "chronic backache" OR "chronic discogenic pain" OR "chronic dorsalgia" OR "chronic lumbago" OR "musculo-skeletal pain" OR "chronic nociceptive pain" OR "chronic visceral pain" OR "chronic musculoskeletal pain" OR "chronic headache" OR "chronic neck pain" OR "chronic shoulder pain" OR "chronic non-specific low back pain")

#2 TS=("neuropsychiatric symptoms" OR "neuropsychiatric syndrome" OR "neuropsychiatric" OR "neuropsychiatric features" OR "neuropsychiatric disorders" OR "neuropsychiatric condition" OR "neurology" OR "psychiatry" OR "neuropsychiatry")

#3 #1 AND #2

**Search Strategy for Scopus**

TITLE-ABS-KEY("chronic pain" OR "chronic primary pain" OR "chronic primary visceral pain" OR "chronic widespread pain" OR "chronic primary musculoskeletal pain" OR "chronic primary headache" OR "chronic primary orofacial pain" OR "chronic migraine" OR "chronic tension-type headache" OR "complex regional pain syndrome" OR "chronic cancer related pain" OR "chronic cancer pain" OR "chronic post cancer treatment pain" OR "chronic postsurgical pain" OR "chronic post traumatic pain" OR "chronic secondary musculoskeletal pain" OR "chronic secondary visceral pain" OR "chronic neuropathic pain" OR "chronic central neuropathic pain" OR "chronic peripheral neuropathic pain" OR "chronic secondary headache" OR "chronic secondary orofacial pain" OR "chronic dental pain" OR "chronic neuropathic orofacial pain" OR "persistent headache" OR "unspecified chronic pain" OR "fibromyalgia" OR "intractable pain" OR "chronic neck and shoulder pain" OR "chronic back pain" OR "chronic pelvic pain" OR "chronic pelvic pain syndrome" OR "chronic low back pain" OR "chronic spine pain" OR "chronic limb pain" OR "urologic chronic pelvic pain syndrome" OR "persistent postoperative pain" OR "widespread chronic pain" OR "chronic orofacial pain" OR "persistent pain" OR "continuous pain" OR "recurrent pain" OR "chronic backache" OR "chronic discogenic pain" OR "chronic dorsalgia" OR "chronic lumbago" OR "musculo-skeletal pain" OR "chronic nociceptive pain" OR "chronic visceral pain" OR "chronic musculoskeletal pain" OR "chronic headache" OR "chronic neck pain" OR "chronic shoulder pain" OR "chronic non-specific low back pain") AND TITLE-ABS-KEY("neuropsychiatric symptoms" OR "neuropsychiatric syndrome" OR "neuropsychiatric" OR "neuropsychiatric features" OR "neuropsychiatric disorders" OR "neuropsychiatric condition" OR "neurology" OR "psychiatry" OR "neuropsychiatry")

**Search Strategy for PubMed**

#1 "chronic pain"[Title/Abstract] OR "chronic primary pain"[Title/Abstract] OR "chronic primary visceral pain"[Title/Abstract] OR "chronic widespread pain"[Title/Abstract] OR "chronic primary musculoskeletal pain"[Title/Abstract] OR "chronic primary headache"[Title/Abstract] OR "chronic primary orofacial pain"[Title/Abstract] OR "chronic migraine"[Title/Abstract] OR "chronic tension-type headache"[Title/Abstract] OR "complex regional pain syndrome"[Title/Abstract] OR "chronic cancer related pain"[Title/Abstract] OR "chronic cancer pain"[Title/Abstract] OR "chronic post cancer treatment pain"[Title/Abstract] OR "chronic postsurgical pain"[Title/Abstract] OR "chronic post traumatic pain"[Title/Abstract] OR "chronic secondary musculoskeletal pain"[Title/Abstract] OR "chronic secondary visceral pain"[Title/Abstract] OR "chronic neuropathic pain"[Title/Abstract] OR "chronic central neuropathic pain"[Title/Abstract] OR "chronic peripheral neuropathic pain"[Title/Abstract] OR "chronic secondary headache"[Title/Abstract] OR "chronic secondary orofacial pain"[Title/Abstract] OR "chronic dental pain"[Title/Abstract] OR "chronic neuropathic orofacial pain"[Title/Abstract] OR "persistent headache"[Title/Abstract] OR "unspecified chronic pain"[Title/Abstract] OR "fibromyalgia"[Title/Abstract] OR "intractable pain"[Title/Abstract] OR "chronic neck and shoulder pain"[Title/Abstract] OR "chronic back pain"[Title/Abstract] OR "chronic pelvic pain"[Title/Abstract] OR "chronic pelvic pain syndrome"[Title/Abstract] OR "chronic low back pain"[Title/Abstract] OR "chronic spine pain"[Title/Abstract] OR "chronic limb pain"[Title/Abstract] OR "urologic chronic pelvic pain syndrome"[Title/Abstract] OR "persistent postoperative pain"[Title/Abstract] OR "widespread chronic pain"[Title/Abstract] OR "chronic orofacial pain"[Title/Abstract] OR "persistent pain"[Title/Abstract] OR "continuous pain"[Title/Abstract] OR "recurrent pain"[Title/Abstract] OR "chronic backache"[Title/Abstract] OR "chronic discogenic pain"[Title/Abstract] OR "chronic dorsalgia"[Title/Abstract] OR "chronic lumbago"[Title/Abstract] OR "musculo-skeletal pain"[Title/Abstract] OR "chronic nociceptive pain"[Title/Abstract] OR "chronic visceral pain"[Title/Abstract] OR "chronic musculoskeletal pain"[Title/Abstract] OR "chronic headache"[Title/Abstract] OR "chronic neck pain"[Title/Abstract] OR "chronic shoulder pain"[Title/Abstract] OR "chronic non-specific low back pain"[Title/Abstract]

#2 "neuropsychiatric symptoms"[Title/Abstract] OR "neuropsychiatric syndrome"[Title/Abstract] OR "neuropsychiatric"[Title/Abstract] OR "neuropsychiatric features"[Title/Abstract] OR "neuropsychiatric disorders"[Title/Abstract] OR "neuropsychiatric condition"[Title/Abstract] OR "neurology"[Title/Abstract] OR "psychiatry"[Title/Abstract] OR "neuropsychiatry"[Title/Abstract]

#3 #1 AND #2

Supplementary Table 1. Quantitatively defined publication-growth phases based on segmented linear regression.

| Phase | Years | Total publications | Mean annual publications | Estimated slope, articles/year |
| --- | --- | --- | --- | --- |
| Phase I | 1983–2000 | 240 | 13.3 | 1.6 |
| Phase II | 2001–2017 | 1405 | 82.6 | 6.3 |
| Phase III | 2018–2020 | 582 | 194.0 | 31.5 |
| Phase IV | 2021–2025 | 1400 | 280.0 | 17.6 |
| Note: Segmented linear regression identified three transition points in annual publication output: 2000, 2017, and 2020. Estimated slopes indicate the fitted annual increase in publication output within each phase. | | | | |

Supplementary Figure S1. Bradford’s Law analysis of source journals.
(A) Top 10 journals ranked by publication output in the Bradford’s Law analysis. (B) Bradford distribution curve showing the cumulative number of publications across source journals. The black solid line represents the observed cumulative article count across the log-ranked journals, and the red dashed line represents the fitted Bradford trend. The journals were divided into three Bradford zones: the core zone (66 sources), Zone 2 (333 sources), and Zone 3 (1049 sources).
